# Artificial intelligence-enabled non-invasive quantification of left ventricular end-systolic pressure: an in silico analysis

**DOI:** 10.1186/s12872-026-06358-8

**Published:** 2026-08-04

**Authors:** Edouard Long, Roberta Dunn, Vitaliy Androshchuk

**Affiliations:** Faculty of Life Sciences and Medicine, King’s College London, London, UK

**Keywords:** Left ventricle, Blood pressure, Artificial intelligence

## Abstract

**Background:**

Invasive pressure–volume loop (PVL) analysis is the gold standard method to quantify myocardial physiology. To enhance broader clinical applicability, non-invasive approaches to derive left ventricular (LV) PVLs have gained prominence, typically estimating LV end-systolic pressure (ESP) from brachial blood pressure (BP) alone. However, this approach does not account for factors that may modulate the pressure waveform as it propagates from the LV to the brachial artery, notably arterial stiffness. We trained an artificial-intelligence (AI) model to predict invasive LVESP from a set of non-invasive hemodynamic parameters and compared its performance with commonly used equations for estimating LVESP using solely brachial BPs.

**Methods:**

An in silico cohort of healthy adults (*N* = 3,837, age: 48.6 ± 17.0y), previously shown to have good agreement for aortic and brachial BP with in vivo data, was split into training and validation cohorts using an internal hold-out validation approach. Clinically relevant parameters were selected as inputs to a random forest machine learning model.

**Results:**

The best performing model included systolic and diastolic brachial BP, heart rate, age, and carotid-femoral pulse wave velocity (PWV), and reliably predicted LVESP (root mean square error: 0.99 mmHg). AI-derived LVESP correlated more strongly with simulated invasive LVESP (r = 0.994) than commonly used equations (r = 0.909–0.908). Bland–Altman analysis demonstrated significant bias in brachial BP-only equations (mean bias: -4.01, limits of agreement [LoA] [± 1.96 standard deviations]: -11.1 to 3.06 mmHg), with the direction of bias varying across different ages. AI-derived LVESP displayed smaller bias (mean bias: 0.002, LoA: -1.94 to 1.94 mmHg) and had negligible age-related bias across the lifespan.

**Conclusions:**

In this hypothesis generating study, a novel non-invasive, AI-enabled approach to LVESP quantification incorporating age, PWV and other key hemodynamic parameters alongside brachial BPs yielded smaller average error across the lifespan than commonly used brachial BP-only equations. These results warrant validation in vivo to ascertain real-world predictive performance.

**Supplementary Information:**

The online version contains supplementary material available at https://doi.org/10.1186/s12872-026-06358-8.

## Introduction

Invasive pressure–volume loop (PVL) analysis is considered the gold standard method to quantify ventricular physiology. Given the inherent challenges of invasive left ventricular (LV) catheterization for PVL quantification, non-invasive methods using echocardiography and magnetic resonance imaging have gained prominence and been validated against invasive measurements [1, 2].

In order to derive the end-systolic pressure–volume relationship and effective arterial elastance (E_a_), end-systolic pressure (ESP) is required. In non-invasive approaches, LVESP is usually estimated from brachial blood pressure (BP) cuff measurements using equations described by Kelly et al*.* in 1992, which were validated in only 10 subjects [3]. Furthermore, this method does not account for key factors which may affect pressure waveform propagation from the LV to the brachial artery, notably the stiffness of the arterial segment, which naturally increases with age [4].

We hypothesized that incorporating a measure of arterial stiffness would improve LVESP prediction across the lifespan. Thus, we trained an artificial intelligence (AI) model to predict LVESP from brachial BP alongside other key hemodynamic parameters, including carotid-femoral pulse wave velocity (cfPWV), and compared its performance with currently used LVESP estimation equations.

## Methods

### Dataset

An in silico dataset of physiologically plausible, healthy patients spread across the lifespan was used for this analysis [5]. This comprised simulated aortic and brachial pressure waveforms (PWs) and derived indices, which were previously validated with in vivo data and showed good agreement alongside reliably reproducing age-related changes in hemodynamic parameters and PW morphology. Physiologically plausible individuals were retained by excluding subjects (*N* = 537) with simulated aortic or brachial BPs outside the 99% age-specific confidence intervals of reference healthy values [6]. Simulated invasive LVESP (LVESP_sim_) was quantified as the pressure at the dicrotic notch of the aortic root PW, as previously described [7], corresponding to aortic valve closure and the end of LV ejection. This approach has been validated as an approximation of the LV end-systolic pressure–volume relationship [8, 9]. Ethical approval for this study was not required as the dataset is publicly available and does not contain any patient-identifiable information.

### Random forest model development

A random forest regression model was selected as the primary learner. Six non-invasively acquired variables were considered as candidate predictors: brachial systolic BP (SBP), brachial diastolic BP (DBP), cfPWV, heart rate (HR), pulse pressure (PP), and age. An internal hold-out validation approach within the single synthetic dataset was used, with 20% of data withheld from model training. Within each candidate model, the development dataset was split 80:20 into training and test sets using the support point method with a sequential nearest neighbor algorithm [10]. Hyperparameters were tuned using a grid search approach with threefold cross validation over the training subset, optimizing the coefficient of determination (R^2^), over the following parameters: number of trees (100,200), number of features considered at each split (square root, or base-2 logarithm of the predictor count – to encourage decorrelation among trees), maximum tree depth [10, 11], minimum samples required to split an internal node [2, 5], and minimum samples per leaf [1, 2]. The latter two parameters were specified in this manner to assess whether limiting tree complexity improved model performance. This produced 32 hyperparameter combinations, each validated across three folds. Subsequently, the best performing hyperparameter configuration identified through cross-validation was then refitted and evaluated on the held-out 20% test subset. All non-empty subsets of the six candidate predictors were evaluated using the fully tuned random forest pipeline, and the final model was selected as the predictor subset that minimized the root mean square error (RMSE) on the held-out test subset. Feature importance in the final model was evaluated using permutation importance with 30 iterations, quantified as the decrease in R^2^ after randomly shuffling the values of each feature. Values predicted in the validation cohort using this methodology are denoted LVESP_RF_. Model training, testing, and validation of LVESP prediction were performed in Python (version 3.13).

### Development of a closed-form equation

To provide a transparent and readily applicable comparison with the random forest model, least absolute shrinkage and selection operator (LASSO) regression was fitted (⍺ = 0.1) to the standardized predictors identified in the final random forest model, using the development dataset. The coefficients were then rescaled to reflect the original variable units and the resulting regularized linear coefficients and intercept were used to derive a closed-form equation for estimating LVESP, termed LVESP_LASSO_. This provides a readily applicable equation that can be used without specialized software.

### LVESP prediction from brachial blood pressure

LVESP was also predicted using the equations described by Kelly et al*.* in the validation cohort [3]:

- 0.9 × SBP (LVESP_0.9xSBP_)
- 2/3 SBP + 1/3 DBP (LVESP_2/3SBP+1/3DBP_)

### Statistical analysis

Variables were assessed for normality using Q-Q plots and quantitatively with the Shapiro–Wilk test. As all variables were normally distributed, continuous data is presented as mean ± standard deviation (SD). Inter-modality agreement was assessed using Bland–Altman analysis, with differences calculated as LVESP_sim_ minus predicted LVESP, and reported as the mean bias with corresponding limits of agreement (LoA), defined as mean bias ± 1.96 SD [12]. Calibration was assessed by regressing LVESP_sim_ on each predicted value. Proportional bias within each Bland–Altman analysis was formally tested by regressing the difference against the mean of the two values. Pearson’s correlation coefficient (r) was reported as a supplementary descriptive statistic. To test for significant differences between correlation coefficients, the Dunn and Clark method was used, which accounts for correlations derived from the same sample. Prediction accuracy was quantified using RMSE and mean absolute error. To formally assess age-related bias, prediction error was regressed against age as a continuous variable for each method. Additionally, a quadratic age term was included to evaluate potential non-linear age effects [13], with its significance assessed using an F-test comparing the linear and quadratic models. Prediction error was additionally characterized across individual age strata. Statistical significance was defined as *P* < 0.05. All analyses were performed using R version 4.4.0 (R Foundation for Statistical Computing, Vienna, Austria).

## Results

Whole cohort characteristics (*N* = 3,837), stratified by age, are presented in Table 1. LVESP_sim_ increased from 95 ± 7 mmHg at age 25 to 107 ± 8 mmHg at age 75. Brachial SBP and cfPWV followed a similar trend, whilst brachial DBP decreased from 72 ± 6 to 69 ± 7 mmHg. Before model development, 767 patients were withheld as the internal hold-out validation cohort, which showed no significant differences with the remaining 3,070 patients in the derivation set (Supplementary Table 1).

**Table 1:** Whole cohort characteristics

Variable	Total (*N* = 3837)	25y (*N* = 712)	35y (*N* = 684)	45y (*N* = 654)	55y (*N* = 641)	65y (*N* = 588)	75y (*N* = 558)
LVESP (mmHg)	102 ± 9	95 ± 7	99 ± 7	104 ± 7	105 ± 7	106 ± 7	107 ± 8
Heart rate (bpm)	76 ± 9	73 ± 9	76 ± 9	77 ± 9	77 ± 9	76 ± 9	74 ± 9
Stroke volume (ml)	60 ± 12	67 ± 13	64 ± 12	61 ± 12	59 ± 11	56 ± 10	54 ± 10
Brachial SBP (mmHg)	118 ± 9	112 ± 9	116 ± 9	120 ± 9	121 ± 9	120 ± 8	120 ± 9
Brachial DBP (mmHg)	73 ± 7	72 ± 6	74 ± 6	76 ± 6	75 ± 6	72 ± 7	69 ± 7
Brachial PP (mmHg)	45 ± 10	40 ± 8	41 ± 9	44 ± 9	46 ± 10	48 ± 10	51 ± 11
Carotid-femoral PWV (m/s)	8.13 ± 1.84	6.31 ± 0.68	6.91 ± 0.87	7.80 ± 0.88	8.53 ± 1.09	9.46 ± 1.41	10.45 ± 1.93

Values are presented as mean ± standard deviation

*Abbreviations*: *DBP* diastolic blood pressure, *LVESP* left ventricular end-systolic pressure, *PP* pulse pressure, *PWV* pulse wave velocity, *SBP* systolic blood pressure

**Fig. 1:**
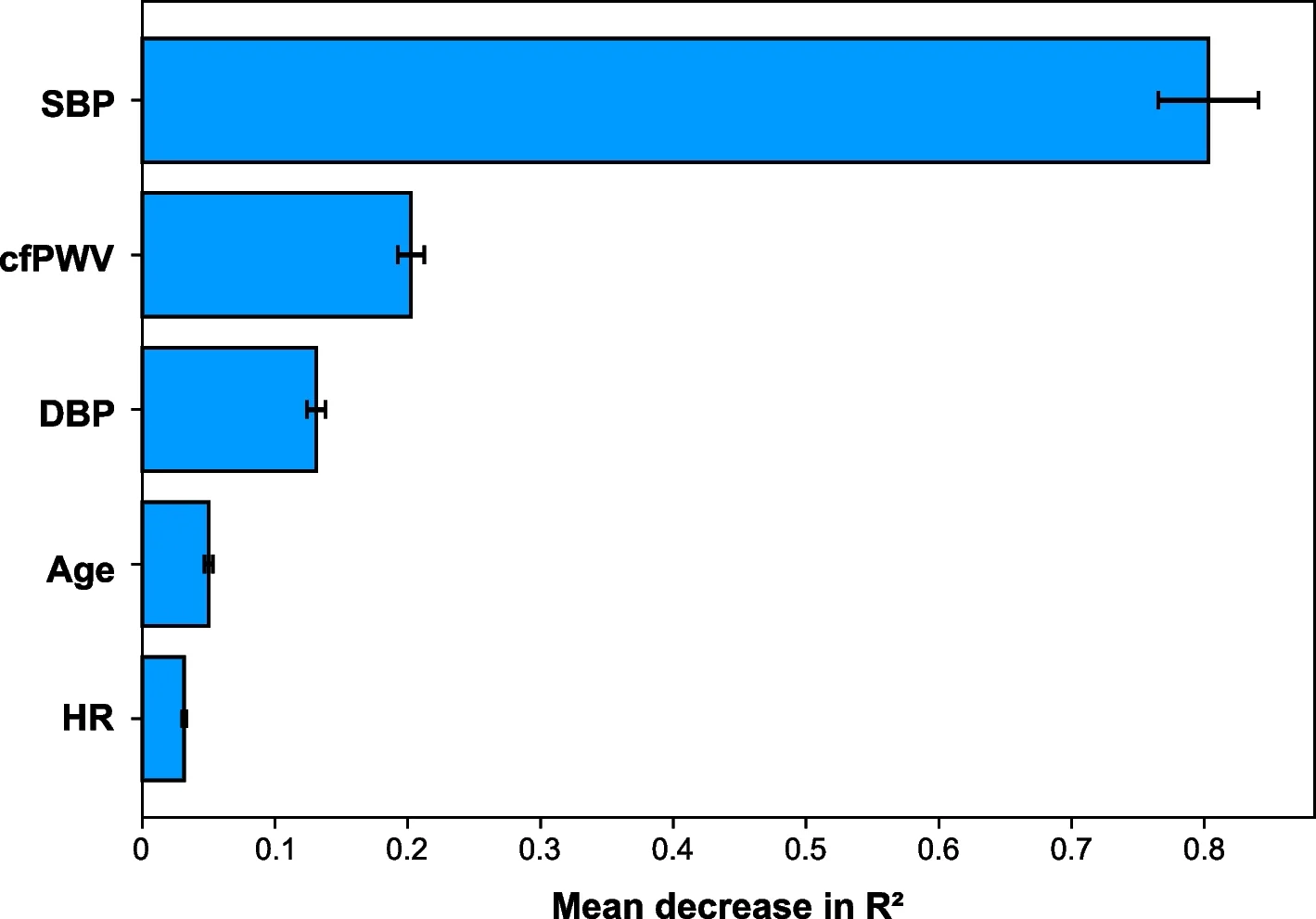
Feature importance in the random forest model. Feature importance is quantified by randomly permuting each predictor and measuring the resulting decrease in model performance (R^2^). Bars represent mean decrease in R.^2^ across 30 permutations and error bars denote the standard deviation. The most important predictor was SBP followed by cfPWV, DBP, age, and HR. Abbreviations: cfPWV: carotid-femoral pulse wave velocity, HR: heart rate. Other abbreviations as in Table 1

The best-performing random forest model (LVESP_RF_) included SBP, DBP, HR, age and cfPWV. Permutation importance (Fig. 1) demonstrated that SBP was the most important feature, followed by cfPWV, DBP, age, and HR. Subsequently, LASSO regression using SBP, DBP, HR, age and cfPWV identified LVESP_LASSO_ = −1.203 + (0.679 × SBP) + (0.327 × DBP) + (−0.165 × HR) + (0.089 × age) + (0.940 × cfPWV). Predicted values are shown in Table 2, with mean LVESP of 102 ± 8 mmHg, 102 ± 8 mmHg, 106 ± 8 mmHg, and 103 ± 7 mmHg for LVESP_RF_, LVESP_LASSO_, LVESP_0.9xSBP_, and LVESP_2/3SBP+1/3DBP_, respectively.

**Table 2:** Predicted values of LVESP in validation cohort

Variable	Total (*N* = 767)	25y (*N* = 140)	35y (*N* = 133)	45y (*N* = 131)	55y (*N* = 125)	65y (*N* = 120)	75y (*N* = 118)
LVESP_sim_ (mmHg)	102 ± 9	94 ± 7	99 ± 7	104 ± 7	105 ± 7	106 ± 7	107 ± 8
LVESP_0.9 x SBP_ (mmHg)	106 ± 8	101 ± 8	104 ± 8	108 ± 8	108 ± 8	108 ± 8	108 ± 8
LVESP_2/3SBP+1/3DBP_ (mmHg)	103 ± 7	99 ± 7	102 ± 7	106 ± 6	105 ± 6	104 ± 6	103 ± 6
LVESP_RF_ (mmHg)	102 ± 8	95 ± 7	99 ± 7	104 ± 7	105 ± 7	106 ± 7	107 ± 8
LVESP_LASSO_ (mmHg)	102 ± 8	95 ± 7	99 ± 7	104 ± 7	105 ± 7	106 ± 7	107 ± 8

Values are presented as mean ± standard deviation

*Abbreviations*: *LVESP*_*LASSO*_ LASSO predicted-LVESP, *LVESP*_*RF*_ random forest-predicted *LVESP* LVESP_sim_: simulated invasive LVESP, *LVESP*_*0.9xSBP*_ LVESP estimated from 0.9 × systolic blood pressure, *LVESP*_*2/3SBP*+*1/3DBP*_ LVESP estimated from 2/3 systolic blood pressure + 1/3 diastolic blood pressure. Other abbreviations as in Table 1

**Fig. 2:**
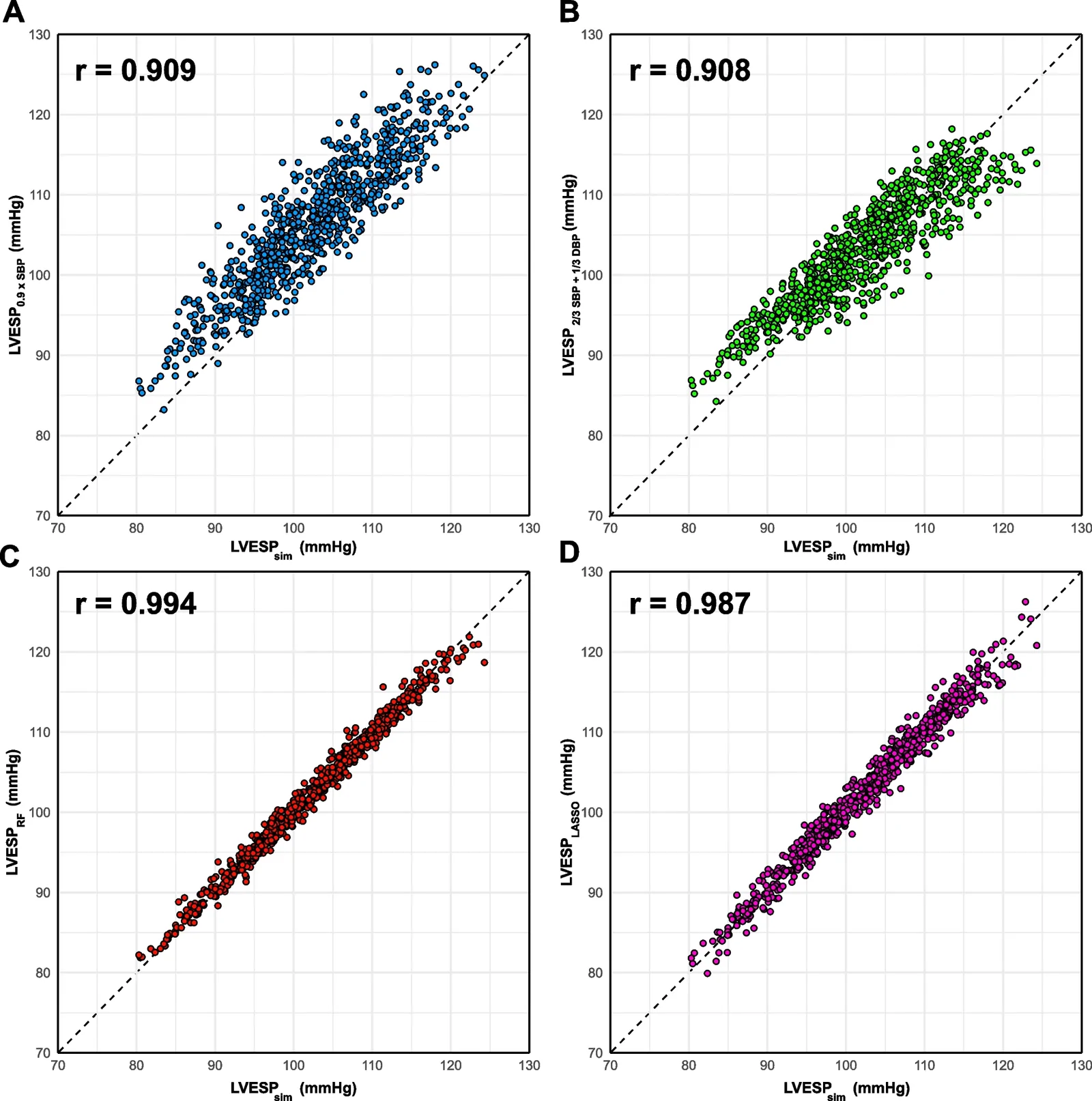
Correlation plots in validation cohort. Plots for correlation between LVESP_sim_ and LVESP_0.9 X SBP_ **A**, LVESP_2/3SBP + 1/3DBP_ **B**, LVESP_RF_ **C**, and LVESP_LASSO_ **D**. Abbreviations as in Table 2

**Fig. 3:**
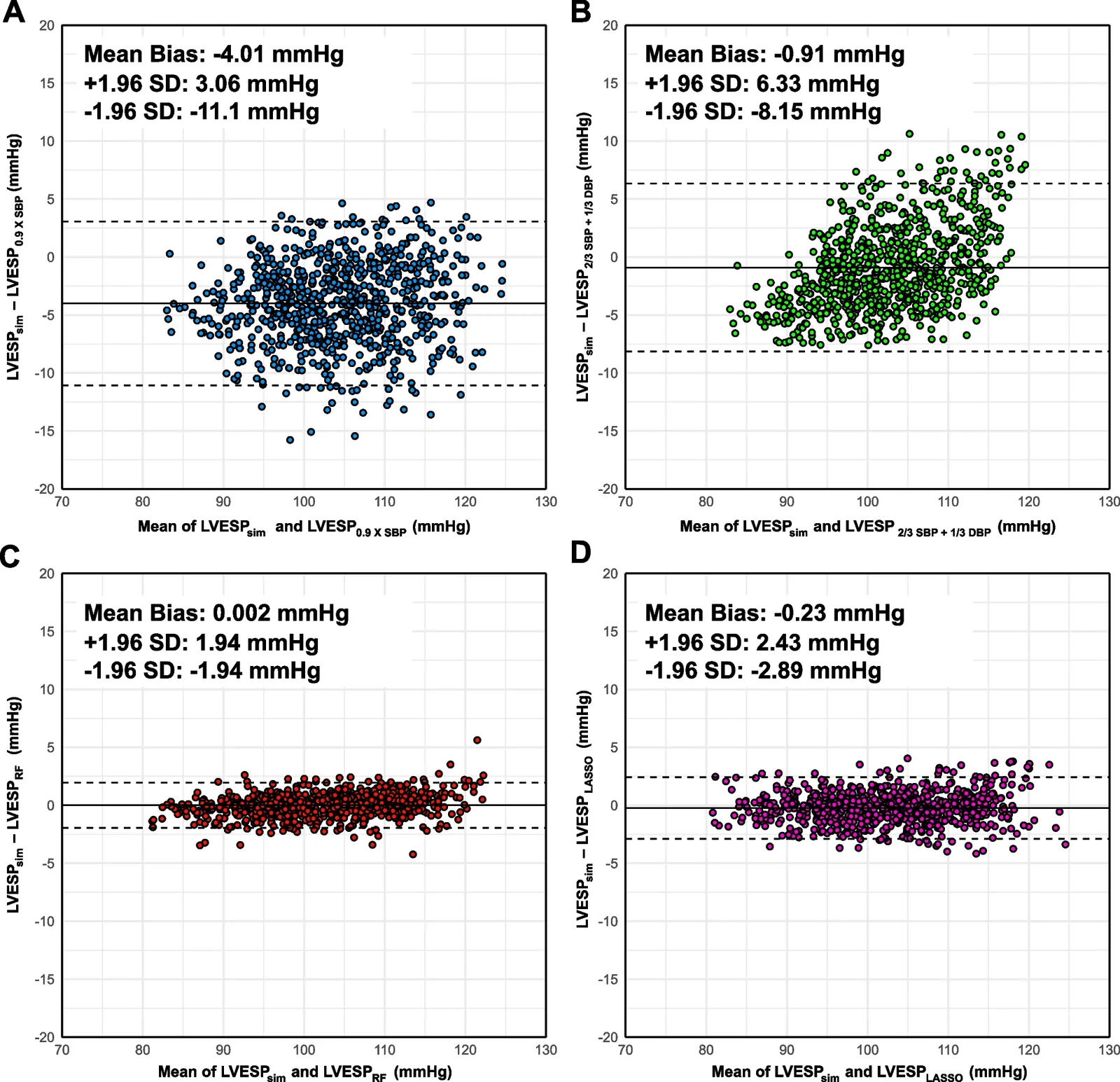
Bland–Altman plots in validation cohort. Bland–Altman plots showing agreement between LVESP_sim_ and LVESP_0.9 X SBP_ **A**, LVESP_2/3SBP + 1/3DBP_ **B**, LVESP_RF_ **C**, and LVESP_LASSO_ **D**. Differences are calculated as LVESP_sim_ minus predicted LVESP. Limits of agreement were defined as ± 1.96 times the standard deviation of the differences. Abbreviations as in Table 2

In terms of prediction accuracy (Table 3, Fig. 2) LVESP_RF_ and LVESP_LASSO_ demonstrated higher correlations (r = 0.99 for both, *p* < 0.001 vs. LVESP_0.9xSBP_ and LVESP_2/3SBP+1/3DBP_), lower RMSE (0.99 mmHg, 1.38 mmHg; respectively), and lower MAE (0.74 mmHg, 1.08 mmHg; respectively), compared to LVESP_0.9xSBP_ (r: 0.91, RMSE: 5.39 mmHg, MAE: 4.43 mmHg), and LVESP_2/3SBP+1/3DBP_ (r: 0.91, RMSE: 3.80 mmHg, MAE: 3.15 mmHg). With respect to agreement with LVESP_sim_ (Fig. 3), LVESP_RF_ demonstrated a mean bias of 0.002 mmHg (LoA: −1.94 to 1.94 mmHg) and LVESP_LASSO_ −0.23 mmHg (LoA: −2.89 to 2.43 mmHg). LVESP_0.9xSBP_ had a greater tendency to underestimate LVESP_sim_ (mean bias: −4.01 mmHg, LoA: −11.1 to 3.06 mmHg), whereas LVESP_2/3SBP+1/3DBP_ showed a smaller underestimation (mean bias: −0.91 mmHg, LoA: −8.15 to 6.33 mmHg). Formal testing for proportional bias (Table 4) demonstrated significant non-zero slopes for all four LVESP estimation methods. The largest bias was observed for LVESP_2/3SBP+1/3DBP_ (slope: 0.22, 95% confidence interval [CI]: 0.19 to 0.26, *p* < 0.001), indicating that prediction error increased substantially at higher LVESP values. LVESP_0.9xSBP_ (slope: 0.04, 95% CI: 0.008 to 0.07, *p* = 0.014), LVESP_RF_ (slope: 0.03, 95% CI: 0.02 to 0.04, *p* < 0.001), and LVESP_LASSO_ (slope: 0.01, 95% CI: 0.002 to 0.02, *p* = 0.025) also demonstrated significant proportional bias, although the magnitude of this effect was smaller.

**Table 3:** Prediction accuracy of LVESP estimation methods in validation cohort

Model	Slope	Intercept	r	RMSE (mmHg)	MAE (mmHg)	Mean Bias (mmHg)	Limits of agreement (mmHg)
LVESP_0.9 x SBP_	0.94	2.03	0.91	5.39	4.43	−4.01	−11.1, 3.06
LVESP_2/3SBP+1/3DBP_	1.13	−13.9	0.91	3.80	3.15	−0.91	−8.15, 6.33
LVESP_RF_	1.02	−2.37	0.99	0.99	0.74	0.002	−1.94, 1.94
LVESP_LASSO_	1.00	−0.24	0.99	1.38	1.08	−0.23	−2.89, 2.43

Limits of agreement were defined as ± 1.96 times the standard deviation of the differences

*Abbreviations*: *RMSE* root mean squared error, *MAE* mean absolute error. Other abbreviations as in Table 2

**Table 4:** Proportional bias of LVESP estimation methods in validation cohort

Model	Slope	95% CI	p-value
LVESP_0.9 x SBP_	0.04	0.008, 0.07	0.014
LVESP_2/3SBP+1/3DBP_	0.22	0.19, 0.26	< 0.001
LVESP_RF_	0.03	0.02, 0.04	< 0.001
LVESP_LASSO_	0.01	0.002, 0.02	0.025

Proportional bias assessed by regressing prediction error (LVESP_sim_ – predicted) against the mean of the two values for each method. Abbreviations as in Table 2

Upon stratification by age group (Table 5), heterogeneous patterns emerged between different LVESP estimation methods. LVESP_RF_ and LVESP_LASSO_ remained strongly correlated with LVESP_sim_ (r ≥ 0.98) across increasing age, with little variation in mean bias. However, while LVESP_0.9xSBP_ retained a broadly strong correlation with LVESP_sim_ (r ≥ 0.90) across age groups, its mean bias showed greater variability, ranging from −6.62 mmHg (LoA: −12.0 to −1.26 mmHg) in 25-year-olds to −1.05 mmHg (LoA: −6.93 to 4.84 mmHg) in 75-year-olds. Similarly, LVESP_2/3SBP+1/3DBP_ showed strong correlation with LVESP_sim_ within individual age groups (r ≥ 0.93), but with greater heterogeneity in mean bias, shifting from under-estimating LVESP_sim_ in 25-year-olds (mean bias: −4.41 mmHg, LoA: −7.63 to −1.19 mmHg) to over-estimating in 75-year-olds (mean bias: 3.73 mmHg, LoA: −2.26 to 9.72 mmHg).

**Table 5:** Prediction accuracy of LVESP estimation methods across individual age groups in validation cohort

Model	Age (years)	r	RMSE (mmHg)	MAE (mmHg)	Mean Bias (mmHg)	Limits of agreement (mmHg)
LVESP_0.9 x SBP_	25y	0.94	7.16	6.62	−6.62	−12.0, −1.26
	35y	0.92	6.29	5.50	−5.41	−11.7, 0.92
	45y	0.91	5.64	4.73	−4.73	−10.8, 1.33
	55y	0.92	4.55	3.71	−3.45	−9.29, 2.40
	65y	0.90	3.99	3.03	−2.12	−8.77, 4.53
	75y	0.93	3.17	2.46	−1.05	−6.93, 4.84
LVESP_2/3SBP+1/3DBP_	25y	0.97	4.70	4.41	−4.41	−7.63, −1.19
	35y	0.96	3.92	3.40	−3.30	−7.47, 0.87
	45y	0.95	3.00	2.53	−1.97	−6.43, 2.50
	55y	0.95	2.32	1.89	−0.53	−4.98, 3.91
	65y	0.93	3.37	2.64	2.01	−3.32, 7.33
	75y	0.93	4.81	3.90	3.73	−2.26, 9.72
LVESP_RF_	25y	0.99	0.96	0.69	−0.19	−2.04, 1.65
	35y	0.99	1.02	0.79	−0.03	−2.03, 1.96
	45y	0.99	0.90	0.73	0.04	−1.72, 1.80
	55y	0.99	0.90	0.68	0.09	−1.69, 1.86
	65y	0.99	0.98	0.76	0.07	−1.86, 2.00
	75y	0.99	1.18	0.83	0.08	−2.23, 2.39
LVESP_LASSO_	25y	0.98	1.30	1.07	−0.17	−2.69, 2.36
	35y	0.98	1.34	1.03	−0.16	−2.77, 2.46
	45y	0.98	1.41	1.10	−0.36	−3.04, 2.32
	55y	0.99	1.20	0.90	−0.28	−2.58, 2.01
	65y	0.98	1.48	1.17	−0.09	−2.99, 2.81
	75y	0.98	1.54	1.21	−0.32	−3.28, 2.65

Limits of agreement were defined as ± 1.96 times the standard deviation of the differences. Abbreviations as in Table 2 and Table 3

In formal analysis of prediction error as a function of age for each LVESP estimation method (Table 6), LVESP_0.9xSBP_ (*p* < 0.001)_,_ LVESP_2/3SBP+1/3DBP_ (*p* < 0.001), and LVESP_RF_ (*p* = 0.016) demonstrated significant linear age-dependent bias. For LVESP_0.9xSBP_, prediction error increased by 0.11 per year of age (95% CI: 0.10 to 0.12), with no evidence of non-linearity, whereas LVESP_2/3SBP+1/3DBP_ demonstrated a steeper linear age effect (slope: 0.17, 95% CI: 0.16 to 0.18) and a significant quadratic term (*p* < 0.001), confirming a non-linear trajectory of age-related bias across the lifespan. Although LVESP_RF_ showed a significant age-related slope (slope: 0.005, 95% CI: 0.001 to 0.009), the magnitude of this effect was substantially smaller than that observed for conventional measures. In contrast, LVESP_LASSO_ showed no significant age-related bias (*p* = 0.633).

**Table 6:** Prediction error as a function of age for LVESP estimation methods in validation cohort

Model	Slope (mmHg/year)	95% CI	p-value (linear)	p-value (quadratic)
LVESP_0.9 x SBP_	0.11	0.10, 0.12	< 0.001	0.451
LVESP_2/3SBP+1/3DBP_	0.17	0.16, 0.18	< 0.001	< 0.001
LVESP_RF_	0.005	0.001, 0.009	0.016	0.165
LVESP_LASSO_	−0.001	−0.007, 0.004	0.633	0.693

Prediction error (LVESP_sim_—predicted LVESP) was regressed on age as a continuous variable. Quadratic p-values are derived from an F-test comparing the linear model to a model incorporating a quadratic age term. Abbreviations as in Table 2

## Discussion

In the present study, we have investigated the potential of a multiparametric AI-enabled approach to predict LVESP using non-invasive hemodynamic measurements. The key findings can be summarized as threefold. First, AI-enabled LVESP estimation incorporating cfPWV, HR, age, and brachial BPs yields highly accurate values, outperforming established and commonly used brachial BP-only methods. Second, the bias observed with LVESP_0.9xSBP_ and LVESP_2/3SBP+1/3DBP_, varied across the lifespan, whereas AI-derived LVESP demonstrated less age-related bias with smaller average error. Third, among the non-AI approaches, LVESP_2/3SBP+1/3DBP_ provides less biased estimates than LVESP_0.9xSBP_.

Since Chen et al*.* first proposed a non-invasive method to derive LV end-systolic elastance using LVESP estimated from brachial BP, non-invasive PVL analysis has been widely applied to study cardiovascular disease including hypertension [14], chronic heart failure [15], myocardial infarction [16], septic shock [17], and valvular heart disease [18]. Notably, many studies continue to estimate LVESP using either LVESP_0.9xSBP_ or LVESP_2/3SBP+1/3DBP_, despite these equations being derived in only 10 patients [3] and externally validated against invasive measurements in just one additional cohort of 17 patients (r = 0.75) [2]. Although numerous studies have validated non-invasive methods to derive LV E_a_ and/or LV E_es_ against invasive measures [1, 2, 19, 20], these have not examined the agreement of the individual components of E_a_ (ESP/SV) or E_es_ (ESP/[ESV-V_0_]). Thus, bias and inaccuracy in the estimation of LVESP specifically cannot be directly inferred. Kappus et al*.* examined LVESP_0.9xSBP_ and LVESP_2/3SBP+1/3DBP_ in a group of 60 healthy patients (mean age 24 ± 0.6 years) and found that neither accurately predicted resting or post-exercise LVESP [11]. However, LVESP was estimated using radial artery applanation tonometry calibrated to brachial BPs, which is known to yield large errors [21] and therefore limits the validity of this study.

In the present study, we found that LVESP_0.9xSBP_ and LVESP_2/3SBP+1/3DBP_ were strongly correlated with LVESP, albeit less so than LVESP_RF_ and LVESP_LASSO_, demonstrating that these simple equations remain broadly valid provided their limitations are considered. Interestingly, Kelly et al*.* reported stronger correlations than observed in our study (r ≥ 0.96), which may be attributable to their younger cohort aged 19–60 years. This is supported by Chen et al*.* who studied an older cohort (62 ± 12 years) and reported a weaker correlation (r = 0.75) between LVESP_0.9xSBP_ and invasive LVESP, consistent with our sub-analyses showing poorer performance of these equations with increasing age. However, it is notable that our AI-enabled approach identified coefficients for brachial SBP (0.679 ≈ 2/3) and DBP (0.327 ≈ 1/3) that are remarkably close to those used in LVESP_2/3SBP+1/3DBP_, further supporting the validity of our method.

The amplification of the arterial pressure pulse wave as it propagates from the LV to the peripheral arteries is a well-established phenomenon in cardiovascular physiology [4]. After leaving the heart, the pulse wave (PW) is reflected throughout the arterial tree. These cumulative reflections combine to form a net reflected wave that propagates both forward and backward. In physiological states without significant stiffening, most reflected waves return to the heart during diastole, thereby augmenting DBP. However, with increasing arterial stiffness and higher PWV, wave reflections may return during systole, reducing DBP augmentation and instead augmenting SBP augmentation and flow pulsatility [22–24]. As a result, LV afterload increases when reflected waves return to the heart during systole rather than diastole, thereby increasing LVESP [22–24]. The magnitude of this amplification is not fixed but diminishes progressively with age as large artery stiffening increases PWV, causing reflected waves to return to the heart during mid-to-late systole, rather than diastole, increasing mid-to-late systolic load and subsequently LVESP. These relationships may be further modified by metabolic processes and disease states, such as diabetes, dyslipidemia, and chronic kidney disease, which can accelerate arterial stiffening beyond the effects of ageing alone. However, these influences could not be captured in our simulated healthy cohort.

In the context of predicting LVESP from brachial BP, it follows that the pressure wave transmission between the ascending aorta and brachial artery should be considered when extrapolating LV pressures from brachial artery measurements. The relevance of this concept is illustrated by the fact that the bias in LVESP_0.9xSBP_, which assumes no contribution of pressure wave amplification, changed linearly with age, consistent with the known linear relationship between age and central-to-peripheral amplification [25]. This is exemplified in our healthy cohort as there are no co-morbidities associated with arterial stiffness that could confound this relationship [26]. Accordingly, the age-related bias in LVESP_2/3SBP+1/3DBP_ did not display this linear association, as DBP begins to decrease from around the sixth decade onwards [27]. Interestingly, all methods demonstrated some degree of proportional bias, although this was substantially greater for LVESP_2/3SBP+1/3DBP_ than for LVESP_0.9xSBP_, LVESP_RF_ and LVESP_LASSO_. This suggests that non-invasive LVESP estimation may be less accurate at higher pressures and that LVESP_2/3SBP+1/3DBP_ should be used with caution in populations with elevated LVESP, such as patients with hypertension.

LVESP_RF_ and LVESP_LASSO_ estimates incorporating cfPWV demonstrated consistently smaller average error across the lifespan and showed stronger overall correlations with LVESP_sim_. However, because the same simulated hemodynamic system was used for both model training and validation, these findings primarily reflect internal consistency and do not necessarily indicate real-world predictive performance. Although brachial BP-only equations remained reasonable surrogates, our findings suggest that a key limitation is their age-dependent bias relative to LVESP. This is particularly notable for LVESP_2/3SBP+1/3DBP_, which shifts from overestimating LVESP in younger adults to underestimating LVESP in older adults. Thus, caution is warranted when applying brachial BP-only equations in cohorts spanning a wide age range, as the associated bias may be unpredictable. As cfPWV may not always be readily available, an appealing approach to improve the accuracy of brachial BP-only equations, without requiring additional parameters, could be to incorporate an age coefficient. This may reduce the unpredictability of bias in these equations and, to some extent, account for differences in arterial stiffness, since age explains approximately half of the variance in cfPWV [28]. However, it should be noted that many other factors and comorbidities influence arterial stiffness [4], and therefore incorporating age into brachial-BP based LVESP prediction will not entirely mitigate inter-individual differences in arterial stiffness.

The potential clinical application of accurately estimating LVESP lies in its ability to improve the prediction of cardiovascular and cerebrovascular events. Although brachial BP has long been recognized as an independent predictor of cardiovascular outcomes, there is evidence that central BP may provide stronger prognostic power [29, 30]. Therefore, routine non-invasive estimation of LVESP using an AI-based multiparametric method may provide additional prognostic power compared with brachial BP or cfPWV alone. Nevertheless, it should be recognized that this dataset is derived from simulated healthy individuals. Therefore, application of this method to individuals with blood pressures outside age-specific healthy reference ranges, or to patients with cardiovascular disease, will require further dedicated study in these populations.

Beyond LVESP estimation, recent techniques that reconstruct the entire LV pressure waveform from peripheral sites warrant discussion. Tamborini et al*.* developed a brachial cuff-based machine learning approach that accurately reconstructed LV pressure waveforms [31]. Although their model showed wider limits of agreement for LVESP (r = 0.89, mean bias: 2.6 mmHg, LoA: −10.4 to 15.5 mmHg) than both brachial BP-only and AI-enabled estimates in the present study, such approaches may enable assessment of additional LV parameters from a single brachial measurement, such as dP/dt, a surrogate index of inotropic state and contractility. Similar approaches have also been described for central aortic pressure waveforms [32, 33]. Nevertheless, it should be considered that these approaches require proprietary software, whereas cohort equations can be applied in clinical practice without specific expertise or software. Future work should aim to reconcile these methods and evaluate their validity across different age groups and disease states.

### Limitations

Several important limitations should be acknowledged. First, given the in silico nature of this study, our findings require validation in real-world patient cohorts and should therefore be considered hypothesis-generating. Moreover, cross-stratification of individuals at distinct age brackets may introduce a risk of data-leakage or optimism. Second, we were unable to assess potential sex-related differences, which may influence both brachial BPs and cfPWV. Third, because the dataset was derived from a healthy population, we were unable to assess the performance of these equations in patients with hypertension. Fourth, we did not have access to an independent external validation cohort and therefore used an internal hold-out validation approach. As a result, both the outcome and predictor variables were generated from the same modeling assumptions. This limited our ability to assess the reliability of the AI models and introduces a risk that they may have learnt the internal mathematical structure of the simulated data rather than a robust, clinically transferable relationship. Thus, the strong correlations and small prediction error observed for the AI models may primarily reflect internal consistency and does not necessarily translate into real-world predictive performance, which will require external in vivo validation. Fifth, data on the timing of the reflected wave were not available, which may influence LVESP. Future studies using brachial BP devices capable of quantifying wave reflection timing, such as SphygmoCor (AtCor Medical, Australia) [34], therefore represent an important area of future research to further refine non-invasive estimation of LVESP. Finally, LVESP estimation from brachial BP may not be applicable in settings where there is a significant pressure gradient between the LV and aorta, such as in aortic stenosis.

## Conclusion

In this hypothesis-generating study of AI-enabled, non-invasive estimation of LVESP, we found that a multiparametric approach incorporating PWV, key hemodynamic parameters and brachial BP predicted LVESP with smaller average error than brachial BP-only approaches. Among brachial BP-only approaches, LVESP_2/3SBP+1/3DBP_ provided more accurate estimates than LVESP_0.9xSBP_. However, both equations exhibited heterogeneous, age-dependent bias, which was not the case for AI-enabled LVESP approaches. These results warrant validation in vivo to ascertain real-world predictive performance.

## Supplementary Information

Supplementary Material 1

## Data Availability

The datasets analyzed during the current study are available at https://doi.org/10.5281/zenodo.3374476.

## Abbreviations

AI: Artificial intelligence
cfPWV: Carotid-femoral pulse wave velocity
DBP: Diastolic blood pressure
E_a_: Effective arterial elastance
E_es_: End-systolic elastance
LASSO: Least absolute shrinkage and selection operator
LV: Left ventricle
LVESP_0.9SBP_: 0.9 × brachial systolic blood pressure
LVESP_2/3SBP+1/3DBP_: 2/3 × brachial systolic blood pressure + 1/3 × brachial diastolic blood pressure
LVESP_LASSO_: LASSO predicted-left ventricular end-systolic pressure
LVESP_RF_: Random forest-predicted left ventricular end-systolic pressure
LVESP_sim_: Simulated invasive left ventricular end-systolic pressure
PVL: Pressure-volume loop
SBP: Systolic blood pressure
SD: Standard deviation
