# Supplementary material for: Artificial intelligence-enabled non-invasive quantification of left ventricular end-systolic pressure: an in silico analysis
Source: BMC Cardiovasc Disord. 2026 Aug 4;26:999. doi: 10.1186/s12872-026-06358-8 (PMC13721338; doi:10.1186/s12872-026-06358-8)
Supplement: Supplementary file 1 [file 12872_2026_6358_MOESM1_ESM.docx]

**Supplementary Table 1:** Comparison of internal-external validation and derivation cohorts

| **Variable** | **Validation cohort (n=767)** | **Derivation cohort (n=3070)** | **p-value** |
| --- | --- | --- | --- |
| LVESP (mmHg) | 102±9 | 102±9 | 0.901 |
| Age (years) |  |  | 0.485 |
| 25 | 140 (18%) | 572 (19%) |  |
| 35 | 133 (17%) | 551 (18%) |  |
| 45 | 131 (17%) | 523 (17%) |  |
| 55 | 125 (16%) | 516 (17%) |  |
| 65 | 120 (16%) | 468 (15%) |  |
| 75 | 118 (15%) | 440 (14%) |  |
| Heart rate (bpm) | 76±9 | 76±9 | 0.539 |
| Stroke volume (ml) | 60±13 | 60±12 | 0.742 |
| Brachial SBP (mmHg) | 118±9 | 118±9 | 0.953 |
| Brachial DBP (mmHg) | 73±7 | 73±7 | 0.956 |
| Brachial PP (mmHg) | 45±10 | 45±10 | 0.986 |
| Carotid-femoral PWV (m/s) | 8.17±1.90 | 8.11±1.82 | 0.465 |

Values are presented as mean ± standard deviation.

**Abbreviations:** DBP: diastolic blood pressure, LVESP: left ventricular end-systolic pressure, PP: pulse pressure, PWV: pulse wave velocity, SBP: systolic blood pressure.
